# First-generation linkage map for the European tree frog (*Hyla arborea*) with utility in congeneric species

**DOI:** 10.1186/1756-0500-7-850

**Published:** 2014-11-26

**Authors:** Christophe Dufresnes, Alan Brelsford, Nicolas Perrin

**Affiliations:** Department of Ecology and Evolution, University of Lausanne, Biophore building, 1015 Lausanne, Switzerland

**Keywords:** Conservation, Heterochiasmy, Hylid frogs, Microsatellites, Population genetics, Recombination, Transcriptome

## Abstract

**Background:**

Western Palearctic tree frogs (*Hyla arborea* group) represent a strong potential for evolutionary and conservation genetic research, so far underexploited due to limited molecular resources. New microsatellite markers have recently been developed for *Hyla arborea*, with high cross-species utility across the entire circum-Mediterranean radiation. Here we conduct sibship analyses to map available markers for use in future population genetic applications.

**Findings:**

We characterized eight linkage groups, including one sex-linked, all showing drastically reduced recombination in males compared to females, as previously documented in this species. Mapping of the new 15 markers to the ~200 My diverged *Xenopus tropicalis* genome suggests a generally conserved synteny with only one confirmed major chromosome rearrangement.

**Conclusions:**

The new microsatellites are representative of several chromosomes of *H. arborea* that are likely to be conserved across closely-related species. Our linkage map provides an important resource for genetic research in European Hylids, notably for studies of speciation, genome evolution and conservation.

**Electronic supplementary material:**

The online version of this article (doi:10.1186/1756-0500-7-850) contains supplementary material, which is available to authorized users.

## Background

Genetic maps based on linkage disequilibrium are powerful tools to address many aspects regarding the evolution of animal genomes like QTL mapping, recombination and chromosome synteny [1]. Moreover, they provide valuable resources for the use of molecular markers in population genetics. European tree frogs (*Hyla arborea* group) have become a model system in this field and were intensively studied in contexts of conservation (e.g. [2]), phylogeography (e.g. [3]), as well as mating systems (e.g. [4]) and sex-chromosome evolution (e.g. [5]). This radiation forms a genetically-rich group, including at least eight species distributed across the Mediterranean Basin, some with deep intraspecific divergences [6]. Nevertheless, despite high potential for research, so far most work has been restricted to the single *H. arborea*, for long the only taxon for which microsatellite markers and their linkage map were available [7]. Indeed, most of these markers turned out to be unusable in congeneric species [8].

To overcome this issue, we have recently developed a new set of EST-derived microsatellite loci with high cross-species utility, thus extending opportunities for research to the entire circum-Mediterranean radiation [8]. Here we present a first-generation linkage map for *H. arborea* combining these new microsatellites with those earlier published, to produce a useful resource for future evolutionary, ecological and conservation genetic applications. As most EST-derived markers could be aligned on the *Xenopus tropicalis* genome [8], we took advantage of this new map to document patterns of synteny of our linkage groups between *Hyla* and *Xenopus*.

## Methods

Tree frog families (parents + tadpoles) were obtained from controlled crosses, as described [9]. Families were chosen from the Balkan Peninsula, where populations display the highest amounts of genetic diversity [3]. Nine families originated from Krk island, Croatia (45.1704°, 14.6229°), one near Karlovac, Croatia (45.5435°, 15.5729°), one near Progar, Serbia (44.7422°, 20.1381°) and one from the Neusiedlersee region, Austria (47.9261°, 16.8634°), for a total of 12 families (n = 352 tadpoles, from 24 to 30 per family). DNA from parents (non-invasive buccal swabs, [10]) and offspring (ethanol fixed tadpoles) was extracted using the Qiagen Robotic Workstation. Our study was approved by the relevant Institutional Animal Care and Use Committee (IACUC), namely the Service de la Consommation et des Affaires Vétérinaires du Canton de Vaud (Epalinges, Switzerland; authorization N°1798) and sampling was conducted under collecting permits (N°532-08-01-01 issued by the Nature Protection Directorate of the Croatian Ministry of Culture; N°353-01-29 issued by the Ministry of Environment and Spatial Planning of the Republic of Serbia); research was carried out in compliance with the Convention on Biological Diversity (CBD) and Convention on the Trade in Endangered Species of Wild Fauna and Flora (CITES); no field-captured animals were harmed and the majority of offspring obtained from the crosses (>80% of each clutch) was released to their ponds of origin.

We genotyped 43 microsatellites polymorphic in at least one parent, including 23 loci mapped by Berset-Brändli et al. [7] (based on Swiss *H. arborea* families) and 20 markers developed since [3, 5, 8, 11]. All but one marker (*Ha*-H116) were amplified in nine multiplex PCRs (Additional file 1: Table S1), following Dufresnes et al. [8]. PCRs were carried out in 10 μL, including 3 μL of DNA (10-100 ng), 3× Qiagen Multiplex Master Mix, and primers (concentrations: Additional file 1: Table S1). Thermal conditions were as follow: 95°C for 15’, 35 × (94°C for 30”, 58°C for 1’30”, 72°C for 1’), 60°C for 30’. Locus *Ha*-H116 was amplified separately in a 10 μL PCR containing 2.5 μL of DNA (10-100 ng), 1× Qiagen PCR buffer (with 1.5 mM of MgCl_2_), 0.2 mM of dNTPs, 0.5 μM of each primer and 0.25 units of Qiagen Taq. Conditions consisted of 94°C for 3’, 45 × (94°C for 45”, 58°C for 45”, 72°C for 1’), 72°C for 5’. PCR products of *Ha*-H116 were pooled and genotyped along with multiplex A. All amplicons were analyzed on an ABI-3100 sequencer with size standards Rox-350 (multiplex A-D) or Rox-500 (multiplex E-I). Alleles were scored with Genemapper 4.0 (Applied Biosystems, Inc.). Additional file 1: Table S1 provides detailed marker information. In complement to our microsatellite dataset, we included genotypes from a SNP within the gene *Fryl*, shown to be sex-linked in *H. arborea* (methods: [11]).

We used CRI-MAP[12] to estimate linkage and recombination rates through calculations of LOD scores (function *twopoint*), determine the most likely order of loci (functions *all* and *flips*) and calculate sex-specific genetic distances (function *build*). The graphical representation of the map was produced with MapChart[13]. We compared recombination rates between Balkanic (this study) and Swiss populations [7] by paired Wilcoxon signed-rank tests, considering only combinations between neighboring informative loci to avoid pseudo-replication.

## Results and discussion

**Figure 1:**
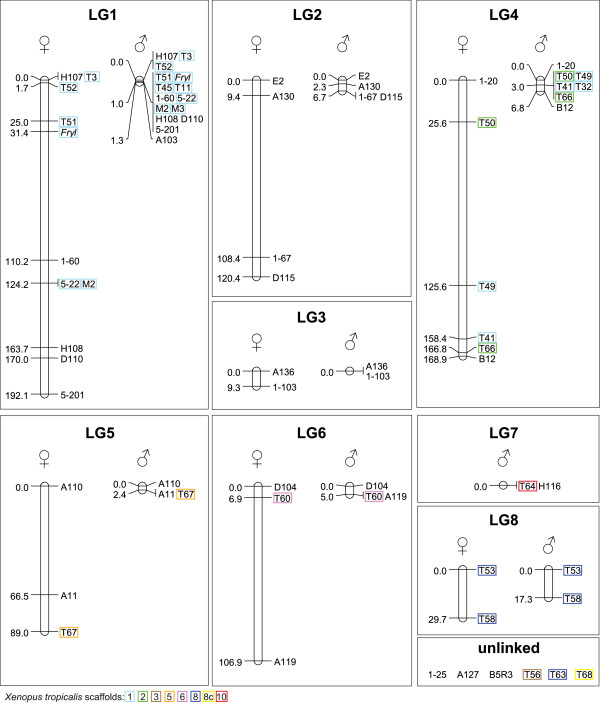
**Male- and female-specific linkage maps for** ***Hyla arborea.*** Maps are based on 43 microsatellites and one SNP locus (*Fryl*), with orders according to the highest log likelihood. For LG7, marker *Ha*-T64 was not polymorphic in females. Genetic distances are indicated in centiMorgan (cM). Colored frames show the location of homologous loci within the *Xenopus tropicalis* genome (assembly 7.1, http://xenbase.org), as reported in [8]. For clarity, simplified marker names were used (see Additional file 1 for full identifiers).

We identified eight linkage groups (Figure 1), potentially representing eight out of the 12 chromosomes of *H. arborea*[14]. LG1 to LG6 corresponds to the six groups reported by Berset-Brändli et al. [7], with LG1 being the sex chromosomes. Two new linkage groups could be identified (LG7 and LG8). Six loci displayed no significant linkage disequilibrium, possibly because of low informativeness and/or because they represent some of the four remaining chromosomes: the same WHA1-25, *Ha*-A127, *Ha*-B5R3 remained similarly unlinked in the Swiss families [7]. However, it is not excluded that several linkage groups map to the same chromosome.

We documented drastically reduced recombination rates in males compared to females across most of the genome, including the sex-linkage group, in accordance with Berset-Brändli et al. [7]. Male recombination seems even under complete arrest for some segments (e.g. LG4: *Ha*-T50 – *Ha*-T66, LG5: *Ha*-A11 – *Ha*-T67). Our autosomal maps were on average 13.7 times longer in females than in males, which is close to what these authors reported for Switzerland (14.3). As they developed, this extreme pattern is in line with the pleiotropic model of Haldane and Huxley [15–17], according to which recombination is repressed over the whole genome in the heterogametic sex (males in *H. arborea*), as a way to prevent recombination between homomorphic sex-chromosomes. Interestingly, however, one linkage group (LG8) does not feature such strong male-biased heterochiasmy, perhaps indicating different mechanisms which would deserve further investigation with additional markers. Moreover, recombination rates did not significantly differ between the two regions, neither in females (Wilcoxon signed rank test, p = 0.26) nor in males (p = 0.87), at least for the few combination of informative loci common to both studies (n = 9 and 12 for females and males respectively). Rates of recombination are provided in Additional file 2: Table S2.

Only one major chromosome rearrangement was apparent since the divergence from *Xenopus* (~200 Mya): *H. arborea*’s LG4 is homologous to both *X. tropicalis*’s scaffold 1 (*Ha*-T32, *Ha*-T41 and *Ha*-T49) and scaffold 2 (*Ha*-T50, *Ha*-T66). Reciprocally, most of *X. tropicalis*’s scaffold 1 is syntenic with *Hyla*’s sex chromosomes (LG1, as shown by [11]), and to this conserved LG4 segment. In contrast, from our data other linkage groups featured no signs of rearrangements: markers lying within different *Xenopus* scaffolds either belong to different *Hyla* linkage groups, or remained unlinked. Accordingly, LG8 includes two markers from the same *Xenopus* scaffold 8. Note that a third marker from this scaffold (*Ha*-T63) remained unlinked, but its low informativeness with other LG8 loci (polymorphic only in one female) prevents firm conclusions. Although the coverage of some chromosomes is weak (i.e. synteny is supported by a few loci per LG), it is thus likely that the same linkage groups hold between *H. arborea* and its Western Palearctic congenerics (diverged over the last 10 My, [6]), and our map should also be suitable for these taxa. High-density linkage mapping using genotyping-by-sequencing (e.g. [18]) will give a much higher resolution for documenting patterns of sex-specific recombination across *H. arborea* chromosomes, and for detecting events of rearrangements across anuran frogs.

The characterization and mapping of new microsatellite markers compatible across Western Palearctic *Hyla* tree frogs (plus some Eastern Palearctic and Nearctic taxa, [8]) significantly expands possibilities for genetic surveys in this group. In particular, their high transferability and known relative genomic localization make them ideal assets for speciation studies, i.e. for analyzing levels of hybridization in secondary contact zones and screening for patterns of differential introgression over the genome. These advantages will also allow multi-species comparative linkage mapping to understand the evolution of sex chromosomes and recombination in this group [5]. Finally, this marker set will be useful to unravel the cryptic diversity suspected in several understudied taxa (e.g. *H. orientalis*, *H. savignyi*, *H. meridionalis*[6]), as well as for other applications related to the conservation of these emblematic species, threatened in many regions and countries.

### Availability of supporting data

Microsatellite genotypes are archived in Dryad (http://doi.org/10.5061/dryad.16pj3). doi:10.5061/dryad.16pj3.

## Electronic supplementary material

Additional file 1: Table S1: Information on the microsatellites used in this study. (DOCX 25 KB)

Additional file 2: Table S2: Sex-specific recombination rates in *Hyla arborea* estimated from Balkanic (this study) and Swiss populations [7]. (DOCX 23 KB)

## Abbreviations

Mya: Million years ago
LG: linkage group.
