# Supplementary material for: First-generation linkage map for the European tree frog (Hyla arborea) with utility in congeneric species
Source: BMC Res Notes. 2014 Nov 26;7:850. doi: 10.1186/1756-0500-7-850 (PMC4258042; doi:10.1186/1756-0500-7-850)
Supplement: Supplementary file 1 — Additional file 1: Table S1: Information on the microsatellites used in this study. (DOCX 25 KB) [file 13104_2014_3369_MOESM1_ESM.docx]

**Additional File 1: Table S1 Information on the microsatellites used in this study.** ref.: reference (listed below); *X.t.*: *Xenopus tropicalis* scaffold; *H.a.*: *Hyla arborea* linkage group (unl.: unlinked); multi.: multiplex.

| **Locus** | **GenBank** | **ref.** | ***X.t.*** | ***H.a.*** | **multi.** | **[primer] (µM)** | **Forward primer** | **Reverse primer** |
| --- | --- | --- | --- | --- | --- | --- | --- | --- |
| ***Ha*-A103** | EU525921 | 3 | - | LG1 | B | 0.50 | F: *HEX*-GCCTAGAAATGTGCAGTGATC | R: CAATTCACACCCAAATCAGAT |
| ***Ha-*A11** | EU029094 | 2 | - | LG5 | C | 0.22 | F: *ATTO*-CCTCCCTCACTCTGCTGAC | R: CAATCCCCGAAAAACATTG |
| ***Ha-*A110** | EU525922 | 3 | - | LG5 | D | 0.30 | F: *ATTO*-AAGGGTTAAATCACCTATCC | R: ACGCAAAAAACATCTGTG |
| ***Ha-*A119** | EU029095 | 2 | - | LG6 | D | 0.15 | F: *ATTO*-CAACTTCCCCCTCTGTTC | R: GCTGAGTGTGAGTGTGTTTG |
| ***Ha-*A127** | EU029096 | 2 | - | unl. | C | 0.30 | F: *HEX*-CTCTGGGTTGCACTACTTAGTC | R: TTCAGGGCTAATTCTTTGTATG |
| ***Ha-*A130** | EU029097 | 2 | - | LG2 | B | 0.12 | F: *FAM*-ATTGCTCACACATACACACAGG | R: GCAGTCACAACTCATTTTGATG |
| ***Ha-*A136** | EU525923 | 3 | - | LG3 | C | 0.20 | F: *HEX*-CCACTGTAAGTAAAATGTGTGC | R: TAAAATCCACCAAGAAACCTAC |
| ***Ha-*B12** | EU029099 | 2 | - | LG4 | A | 0.80 | F: *HEX*-AATGGTATCTCGGTGGTATCC | R: TTGAAAAATCTCTCCCTACAGC |
| ***Ha-*B5R3** | EU029098 | 2 | - | unl. | C | 0.05 | F: *FAM*-CCCCTTTAGAGTCGCCATAC | R: AGCCATCTTGTGGTCAGTCA |
| ***Ha-*D104** | EU525925 | 3 | - | LG6 | D | 0.18 | F: *FAM*-GCTGGCTGACTTATTCTTTG | R: TCTTCTCTCCACGGTCTTC |
| ***Ha*-D110** | EU525927 | 3 | - | LG1 | E | 0.30 | F: *HEX*-AACTGCATGTTCATGTTTCAC | R: CCTGACTTCTTAAATGTGCTTT |
| ***Ha-*D115** | EU029104 | 2 | - | LG2 | D | 0.30 | F: *FAM*-GTTTTTCGATTCCTGGATAAC | R: TGGGAGTTTTCAAAAGTGAC |
| ***Ha-*E2** | EU029103 | 2 | - | LG2 | D | 0.30 | F: *HEX*-ACAACTTCCAACTGGAGTCAAC | R: CCTTAGTGGGAGCTGTAATCAC |
| ***Ha*-H107** | EU029101 | 2 | - | LG1 | E | 0.70 | F: *FAM*-CACCCTGGTAGGGAAATTC | R: GGCAAATGGGGATGAGTA |
| ***Ha*-H108** | EU029102 | 2 | - | LG1 | E | 0.10 | F: *FAM*-GGGGGTGAGTAAGGGTTAAATC | R: GCCACTGTATAGTCCCTCCCTA |
| ***Ha-*H116** | EU525928 | 3 | - | LG7 | - | - | F: *ATTO*-AATGGGGGTGAGTAAGGGTTA | R: CAGGTCCTGACACTGTGACAC |
| ***Ha*-M2** | - | 4 | 1 | LG1 | B | 0.20 | F: *HEX*-GCCTGTTGAGCTGCTTGC | R: GGGCAGTGCAAGCTCAGC |
| ***Ha*-M3** | - | 4 | 1 | LG1 | B | 0.20 | F: *HEX*-CTGGTTTTGCTGTTGCTGAA | R: TCAAGTCACCCAGCAGAATG |
| ***Ha*-T11** | - | 5 | 1 | LG1 | G | 0.10 | F: *FAM*-TGAGGTGGCTGATTGAACTG | R: AACCTGAACCAGCATGGAAC |
| ***Ha*-T3** | - | 5 | 1 | LG1 | F | 0.10 | F: *FAM*-TCTGCCCCATTATTTTCAGC | R: ATGGCATCACGGATTCATTT |
| ***Ha*-T32** | - | 5 | 1 | LG4 | F | 0.20 | F: *HEX*-GTCCACCTCCTAGCTCATGC | R: CTGGAAAATGGCCTGTTCAT |
| ***Ha*-T41** | - | 5 | 1 | LG4 | G | 0.10 | F: *HEX*-AGAATTTGGGGTAGGGGGTA | R: GGCTCAAAGCTGAATGGAAG |
| ***Ha*-T45** | - | 5 | 1 | LG1 | G | 0.20 | F: *FAM*-CGGCACTTCTCCAACAGATT | R: ATGACCACTTCCAGCCTCAG |
| ***Ha*-T49** | - | 5 | 1 | LG4 | F | 0.10 | F: *ATTO*-CCTGAGCAAGCTGAGAAGGA | R: TTGCTTGTTGGTTTCAGTGC |
| ***Ha*-T50** | KF598777 | 6 | 2 | LG4 | H | 0.10 | F: *HEX*-CAGCCCAACTGACTGGTTTT | R: GGGGAAGACTTTGACCCTCA |
| ***Ha*-T51** | - | 5 | 1 | LG1 | F | 0.60 | F: *ATTO*-CATTTCAGGACGTCATGGTG | R: ACCCTATCCAATGGGGAAAA |
| ***Ha*-T52** | - | 5 | 1 | LG1 | F | 0.20 | F: *FAM*-GGAAGTCTGCATTCGCTCAT | R: GAGCGCTGAGCATATACTGG |
| ***Ha*-T53** | - | 7 | 8 | LG8 | H | 0.20 | F: *HEX*-TCTCCTGTCCTTCACCCAAC | R: CTTCCCAGCCTGGAACATC |
| ***Ha*-T56** | KF598778 | 6 | 3 | unl. | H | 0.20 | F: *ATTO*-TGCAAAAATGCCATGAAGTC | R: TTTGGAGACATCACGGTTGA |
| ***Ha*-T58** | KF598779 | 6 | 8 | LG8 | H | 0.10 | F: *HEX*-TCCCGAAAGGACTACTGCTG | R: ACGCACAGGAGGAGAAAGAA |
| ***Ha*-T60** | KF598780 | 6 | 6 | LG6 | I | 0.10 | F: *FAM*-ATTGCGAAAAACTGGTGGTT | R: GCTTTTCCCAGATCAACAGG |
| ***Ha*-T63** | KF598781 | 6 | 8 | unl. | I | 0.10 | F: *ATTO*-TTCTGACCTCTCGGTTTGCT | R: ATGTAAAGGCGCTGATGGAG |
| ***Ha*-T64** | KF598782 | 6 | 10 | LG7 | G | 0.20 | F: *HEX*-CCCCAAGACAGAAGGACATC | R: TACATCACCCGAAGTGCAGA |
| ***Ha*-T66** | KF598783 | 6 | 2 | LG4 | I | 0.10 | F: *FAM*-CTCTTTCGGGTTCCATGCT | R: TCCATTGTGCTGATCGTGTT |
| ***Ha*-T67** | KF598786 | 6 | 5 | LG5 | I | 0.25 | F: *ATTO*-GGGCAGCTTTATTTTTCAGC | R: AGTGGCACCTCCAATAAAGG |
| ***Ha*-T68** | KF598784 | 6 | 8c | unl. | I | 0.10 | F: *HEX*-AGGGCAGAGATACAGGCGTA | R: TGAAACAAATACCGGACTGC |
| **WHA1-103** | AJ403992 | 1 | - | LG3 | B | 0.45 | F: *FAM*-CAAAGTGACAATGTGGGGTCTCAT | R: ATAGCATCAAATCCAGCCGTAGG |
| **WHA1-20** | AJ403986 | 1 | - | LG4 | A | 0.08 | F: *FAM*-GTCCCTTCCTGAATAAGTGTCG | R: CCATTCCCTCCTGGCTTT |
| **WHA1-25** | AJ403987 | 1 | - | unl. | A | 0.50 | F:*FAM*-AAGAATCTGCCGCAAAGAAG | R: TAGGAAGGGACAGGAGGTCA |
| **WHA1-60** | AJ403989 | 1 | - | LG1 | E | 0.40 | F: *ATTO*-TAGGTCATGTATAGCCTGTT | R: TCTGTTTACTTCAGGGGT |
| **WHA1-67** | AJ403991 | 1 | - | LG2 | C | 0.29 | F: *ATTO*-GCTTTACACATGGGGGTAT | R: CACTCCTTTTAGAGTATGTTGTTG |
| **WHA5-201** | AJ403999 | 1 | - | LG1 | E | 0.30 | F: *HEX*-TCATGGACTGTCGTCATGGT | R: AGGTAAATGGAATCTGGGTGTG |
| **WHA5-22** | AJ403996 | 1 | 1 | LG1 | E | 0.10 | F: *ATTO*-TTACAGCAACAGCAAATGG | R: ATCAGGGACTGGGTCTGT |

^1^ Arens P, Van't Westende W, Bugter R, Smulders MJM, Vosman B: Microsatellite markers for the European tree frog *Hyla arborea*. *Mol Ecol* 2000, 9:1944-1946.

^2^ Berset-Brändli L, Jaquiéry J, Broquet T, Perrin N: Isolation and characterization of microsatellite loci for the European tree frog (*Hyla arborea*). *Mol Ecol Resour* 2008, 8:1095-1097.

^3^ Berset-Brändli L, Jaquiéry J, Broquet T, Ulrich Y, Perrin N: Extreme heterochiasmy and nascent sex chromosomes in European tree frogs. *Proc R Soc B* 2008, 275:1577-1585.

^4^ Stöck M, Horn A, Grossen C, Lindtke D, Sermier R, Betto-Colliard C, Dufresnes C, Bonjour E, Dumas Z, Luquet E, Maddalena T, Clavero Sousa H, Martinez-Solano I, Perrin N: Ever-young sex chromosomes in European tree frogs. *PLoS Biol* 2011, 9:e1001062.

^5^ Brelsford A, Stöck M, Betto-Colliard C, Dubey S, Dufresnes C, Jourdan-Pineau H, Rodrigues N, Savary R, Sermier R, Perrin N: Homologous sex chromosomes in three deeply divergent anuran species. *Evolution* 2013, 67:2434-2440.

^6^ Dufresnes C, Wassef J, Ghali K, Brelsford A, Stöck M, Lymberakis P, Crnobrnja Isailović J, Perrin N. Conservation phylogeography: does historical diversity contribute to regional vulnerability in European tree frogs (*Hyla arborea*)? *Mol Ecol* 2013, 22:5669-5684.

^7^ Dufresnes C, Brelsford A, Béziers P, Perrin N: Stronger transferability but lower variability in transcriptomic- than in anonymous microsatellites: evidence from Hylid frogs. *Mol Ecol Resour* 2014, 14:716-725.
