# Supplementary material for: First-generation linkage map for the European tree frog (Hyla arborea) with utility in congeneric species
Source: BMC Res Notes. 2014 Nov 26;7:850. doi: 10.1186/1756-0500-7-850 (PMC4258042; doi:10.1186/1756-0500-7-850)
Supplement: Supplementary file 2 — Additional file 2: Table S2: Sex-specific recombination rates in Hyla arborea estimated from Balkanic (this study) and Swiss populations [7]. (DOCX 23 KB) [file 13104_2014_3369_MOESM2_ESM.docx]

**Additional File 2: Table 2 Sex-specific recombination rates in *Hyla arborea*.** Male and female rates are shown above and below diagonals respectively. Recombination rates estimated from Swiss families are indicated in brackets. Non-informative combinations of markers are noted “na”.

| **LG1** | *Ha*-H107 | *Ha*-T3 | *Ha*-T52 | *Ha*-T51 | *Fryl* | *Ha*-T45 | *Ha*-T11 | WHA1-60 | *Ha*-M2 | *Ha*-M3 | WHA5-22 | *Ha*-H108 | *Ha*-D110 | WHA5-201 | *Ha*-A103 |
| --- | --- | --- | --- | --- | --- | --- | --- | --- | --- | --- | --- | --- | --- | --- | --- |
| *Ha*-H107 | - | 0.00 | na | 0.00 | na | na | na | na (0.00) | na | na | na (0.00) | na (0.00) | na (0.00) | na (0.00) | na (0.00) |
| *Ha*-T3 | 0.00 | - | 0.00 | 0.00 | 0.00 | 0.01 | 0.00 | 0.01 | 0.00 | 0.01 | 0.01 | 0.01 | 0.01 | 0.00 | 0.01 |
| *Ha*-T52 | na | 0.02 | - | 0.01 | na | 0.02 | 0.00 | 0.01 | 0.00 | 0.02 | 0.01 | 0.01 | 0.00 | 0.01 | 0.01 |
| *Ha*-T51 | 0.00 | 0.23 | 0.21 | - | 0.00 | 0.00 | 0.00 | 0.00 | na | 0.00 | 0.00 | 0.00 | 0.00 | 0.00 | 0.00 |
| *Fryl* | na | 0.28 | na | 0.03 | - | 0.00 | na | 0.00 | na | 0.00 | 0.00 | 0.00 | na | 0.00 | 0.00 |
| *Ha*-T45 | na | na | na | na | na | - | na | 0.00 | na | 0.00 | 0.00 | 0.00 | 0.00 | 0.00 | 0.00 |
| *Ha*-T11 | na | na | na | na | na | na | - | 0.00 | 0.00 | na | 0.00 | 0.00 | 0.00 | 0.00 | 0.00 |
| WHA1-60 | na (0.47) | 0.50 | 0.46 | 0.50 | 0.40 | na | na | - | 0.00 | 0.00 | 0.00 (0.00) | 0.00 (0.00) | 0.00 (0.00) | 0.00 (0.00) | 0.00 (0.00) |
| *Ha*-M2 | na | 0.50 | 0.50 | na | na | na | na | 0.23 | - | na | 0.00 | 0.00 | 0.00 | 0.00 | 0.00 |
| *Ha*-M3 | na | na | na | na | na | na | na | na | na | - | 0.00 | 0.00 | 0.00 | 0.00 | 0.00 |
| WHA5-22 | na (na) | 0.49 | 0.50 | 0.50 | 0.41 | na | na | 0.14 (na) | 0.00 | na | - | 0.00 (0.00) | 0.00 (0.00) | 0.00 (0.00) | 0.00 (0.00) |
| *Ha*-H108 | na | 0.47 | na | 0.50 | 0.40 | na | na | 0.39 (na) | na | na | 0.37 (na) | - | 0.00 (0.00) | 0.00 (0.00) | 0.00 (0.00) |
| *Ha*-D110 | na (0.45) | 0.50 | 0.49 | 0.50 | na | na | na | 0.32 (0.50) | 0.33 | na | 0.30 (na) | 0.07 (na) | - | 0.00 (0.00) | 0.00 (0.00) |
| WHA5-201 | na (0.50) | 0.50 | 0.48 | 0.48 | 0.48 | na | na | 0.50 (0.50) | 0.37 | na | 0.43 (na) | 0.28 (na) | 0.20 (0.14) | - | 0.01 (0.00) |
| *Ha*-A103 | na (na) | na | na | na | na | na | na | na (na) | na | na | na (na) | na (na) | na (na) | na (na) | - |

| **LG2** | *Ha*-E2 | *Ha*-A130 | WHA1-67 | *Ha*-D115 |
| --- | --- | --- | --- | --- |
| *Ha*-E2 | - | 0.06 (0.08) | 0.07 (0.14) | 0.07 (0.09) |
| *Ha*-A130 | 0.08 (0.05) | - | 0.05 (0.01) | 0.04 (0.02) |
| WHA1-67 | 0.33 (0.31) | 0.50 (0.41) | - | 0.00 (0.01) |
| *Ha*-D115 | 0.49 (0.21) | 0.50 (0.38) | 0.12 (0.15) | - |

| **LG3** | *Ha*-A136 | WHA1-103 |
| --- | --- | --- |
| *Ha*-A136 | - | 0.00 (0.03) |
| WHA1-103 | 0.09 (0.08) | - |

| **LG4** | WHA1-20 | *Ha*-T50 | *Ha*-T49 | *Ha*-T41 | *Ha*-T66 | *Ha*-T32 | *Ha*-B12 |
| --- | --- | --- | --- | --- | --- | --- | --- |
| WHA1-20 | - | 0.00 | 0.04 | 0.05 | 0.09 | 0.00 | 0.02 (0.00) |
| *Ha*-T50 | 0.25 | - | 0.00 | 0.00 | 0.00 | na | 0.07 |
| *Ha*-T49 | 0.49 | 0.50 | - | 0.00 | 0.00 | na | 0.04 |
| *Ha*-T41 | 0.50 | 0.49 | 0.25 | - | 0.00 | 0.00 | 0.03 |
| *Ha*-T66 | 0.49 | 0.50 | 0.30 | 0.09 | - | na | 0.10 |
| *Ha*-T32 | na | na | na | na | na | - | 0.00 |
| *Ha*-B12 | 0.50 (0.49) | 0.43 | 0.33 | 0.05 | 0.02 |  | - |

| **LG5** | *Ha*-A110 | *Ha*-A11 | *Ha*-T67 |
| --- | --- | --- | --- |
| *Ha*-A110 | - | 0.03 (0.01) | 0.03 |
| *Ha*-A11 | 0.49 (0.37) | - | 0.00 |
| *Ha*-T67 | 0.46 | 0.22 | - |

| **LG6** | *Ha*-D104 | *Ha*-T60 | *Ha*-A119 |
| --- | --- | --- | --- |
| *Ha*-D104 | - | 0.00 | 0.05 (0.05) |
| *Ha*-T60 | 0.08 | - | 0.00 |
| *Ha*-A119 | 0.50 (0.44) | 0.50 | - |

| **LG7** | *Ha*-H116 | *Ha*-T64 |
| --- | --- | --- |
| *Ha*-H116 | - | 0.00 |
| *Ha*-T64 | na | - |

| **LG8** | *Ha*-T53 | *Ha*-T58 |
| --- | --- | --- |
| *Ha*-T53 | - | 0.17 |
| *Ha*-T58 | 0.27 | - |
